# Maipomycin A, a Novel Natural Compound With Promising Anti-biofilm Activity Against Gram-Negative Pathogenic Bacteria

**DOI:** 10.3389/fmicb.2020.598024

**Published:** 2021-01-12

**Authors:** Junliang Zhang, Xiaoyan Liang, Shiling Zhang, Zhiman Song, Changyun Wang, Ying Xu

**Affiliations:** ^1^Shenzhen Key Laboratory of Marine Bioresource and Eco-Environmental Science, Shenzhen Engineering Laboratory for Marine Algal Biotechnology, College of Life Sciences and Oceanography, Shenzhen University, Shenzhen, China; ^2^Key Laboratory of Optoelectronic Devices and Systems of Ministry of Education and Guangdong Province, College of Physics and Optoelectronic Engineering, Shenzhen University, Shenzhen, China; ^3^Key Laboratory of Marine Drugs, Ministry of Education of China, School of Medicine and Pharmacy, Ocean University of China, Qingdao, China; ^4^Laboratory for Marine Drugs and Bioproducts, Qingdao National Laboratory for Marine Science and Technology, Qingdao, China; ^5^Institute of Evolution and Marine Biodiversity, Ocean University of China, Qingdao, China

**Keywords:** maipomycin A, biofilm inhibition, Gram-negative bacteria, iron chelator, antibiotics enhancer

## Abstract

Pathogenic bacterial biofilms play an important role in recurrent nosocomial and medical device-related infections. Once occurred, the complex structure of the biofilm promotes the development of antibiotic resistance and becomes extremely difficult to eradicate. Here we describe a novel and effective anti-biofilm compound maipomycin A (MaiA), which was isolated from the metabolites of a rare actinomycete strain *Kibdelosporangium phytohabitans* XY-R10. Its structure was deduced from analyses of spectral data and confirmed by single-crystal X-ray crystallography. This natural product demonstrated a broad spectrum of anti-biofilm activities against Gram-negative bacteria. Interestingly, the addition of Fe(II) or Fe(III) ions could block the biofilm inhibition activity of MaiA because it is an iron chelator. However, not all iron chelators showed biofilm inhibition activity, suggesting that MaiA prevents biofilm formation through a specific yet currently undefined pathway. Furthermore, MaiA acts as a synergist to enhance colistin efficacy against *Acinetobacter baumannii*. Our results indicate that MaiA may potentially serve as an effective antibiofilm agent to prevent Gram-negative biofilm formation in future clinical applications.

## Introduction

Bacterial resistance against antibiotics is now a global health problem of increasing importance, one of the major mechanisms of resistance is the formation of biofilms by pathogens. Biofilms are sessile microbial communities embedded in self-produced polymeric substances (polysaccharide, protein (polysaccharides, proteins, and DNA), which help microbes colonization of the surface of human organs or medical devices (Flemming and Wingender, 2010). The biofilms act as effective barriers to prevent drugs permeation and alter the physiological states of bacteria (Hall and Mah, 2017). Consequently, bacterial cells of biofilms possess more resistant (10–1,000 times) to antibiotics compared with planktonic forms (Stewart and Costerton, 2001).

It has been reported that biofilms contribute over 80% of human pathogen infections (Pandin et al., 2017). The biofilms of “ESKAPE” pathogens (*Enterococcus faecalis*, *Staphylococcus aureus*, *Klebsiella pneumoniae*, *Acinetobacter baumannii*, *Pseudomonas aeruginosa*, and *Enterobacter* spp.) commonly present the most acute threat (Ma et al., 2020). Of particular concerns are the emergence of Gram−negative bacteria (GNB) like *A. baumannii*, *P. aeruginosa*, *K. pneumoniae*, and *Enterobacter* spp., which are often recalcitrant to “the last line of antibiotics” such as carbapenems and colistin (Smith et al., 2018; Luther et al., 2019). In recent years, nosocomial infections caused by biofilm forming GNB such as pneumonia, urinary tract infections, endocarditis, wound infection, and bacteremia, are becoming increasingly deadly (Lyczak et al., 2000; Dijkshoorn et al., 2007; Prowle et al., 2011; Frykberg and Banks, 2015). In particular, ventilator-associated pneumonia with a mortality rate as high as 60% (Bertolini et al., 2018), and catheter-associated urinary tract infections with morbidity rate approximately to 70% (Shuman and Chenoweth, 2018) are resulted from biofilm-related infections mainly caused by *A. baumannii*, *P. aeruginosa*, and *Enterobacter* spp.

Currently, antibiotic treatment is the most important and effective way to control microbial infections and prevent biofilm formation (Bjarnsholt et al., 2018). However, it is almost impossible to eradicate biofilm infections with antibiotics *in vivo* (Wu et al., 2015). Therefore, the necessity to find effective drugs that could inhibit and eliminate biofilms is an important attention. Based on the advancements of biofilm formation mechanism, numerous anti-biofilm molecules are discovered with different action modes such as inhibit quorum sensing, hinder cell adhesion, disperse extracellular polymeric substance and interfere with c-di-GMP signaling pathways, and so on (Roy et al., 2018). The natural products have greater structural and biochemical diversity compared with synthetic compounds (Genilloud, 2017), thus it is useful in the development of anti-biofilm agents. Recently, there have been reports about a variety of bacterial products including small molecules, enzymes, exopolysaccharides, and peptides isolated possessing anti-biofilm activities against different pathogens (Khan et al., 2019). Most of these studies have only studied the biofilm inhibition effect against a single species or genus of bacteria, such as *P. aeruginosa*, *Escherichia coli*, or *Staphylococcus*. Yet only very few natural products like exopolysaccharides showed broad-spectrum anti-biofilm effects (Valle et al., 2006; Jiang et al., 2011; Sayem et al., 2011; Mahdhi et al., 2017).

In our continuing effort to screen microbial natural products against biofilms, we identified a non-toxic, effective and broad-spectrum anti-biofilm compound butanolide derived from a marine *Streptomyces* sp. in previous work (Yin et al., 2019). Here we report the isolation and characterization of a new compound maipomycin A (MaiA) derived from a mangrove rare actinomycete strain. We then evaluated its anti-biofilm efficiency against several pathogenic bacteria to inquire its broad-spectrum anti-biofilm ability. In addition, we investigated the possible mode of action of MaiA, and compared the biofilm inhibition activity with its two analogs to analyze their structure-activity relationships. Finally, the synergistic antibacterial and anti-biofilm effect between MaiA with antibiotics was also examined.

## Materials and Methods

### Strains and Chemicals

Gram-negative and Gram-positive bacterial strains including *A. baumannii* ATCC 19606, *P. aeruginosa* ATCC 27853, *E. coli* ATCC 25922, *K. pneumoniae* ATCC 13883, methicillin-sensitive *S. aureus* ATCC 25923 (MSSA), methicillin-resistant *S. aureus* ATCC 43300 (MRSA), and several clinical strains of multidrug-resistant were kindly provided by Prof. Dai-Jie Chen from the Shanghai Jiao Tong University. Chemicals, media, and antibiotics were purchased from Sigma-Aldrich (Poole, United Kingdom) unless otherwise stated. Collismycin A and other two analogs were obtained from Alfa Chemistry (New York, United States) and Shenzhen GenProMetab Biotechnology Company.

### Bioassay-Guided Isolation and Structure Determination of the Anti-biofilm Compound

The strain *Kibdelosporangium phytohabitans* XY-R10 was isolated from the root sediments (3–5 cm) of a mangrove plant *Kandelia candel* (L.) Druce, collected from Mai Po Inner Deep Bay Ramsar Site (E 114.05°, N22.49°; Hong Kong, China). The bacterium was cultivated in multiple 250 mL Erlenmeyer flasks each containing 80 mL of SGTPY medium (5 *g* starch, 5 *g* glucose, 1 *g* tryptone, 1 *g* peptone, 1 *g* yeast extract, and 17 *g* sea salts dissolved in 1 L of distilled water) at 28°C with agitation of 200 rpm for 6 days. The culture broth (10 L) was extracted with a double volume of ethyl acetate (EtOAc) three times. The combined EtOAc layers were dried by an evaporator to give a total crude extract (1.5 *g*), which was then applied to ODS column eluted with a step-gradient of water, (4:1) water/methanol (MeOH), (3:2) water/MeOH, (2:3) water/MeOH, (1:4) water/MeOH, and 100% MeOH, yielding six fractions. The fractions were tested in algicidal bioassays and the active fraction (4:1) water/MeOH was separated further using a Phenomenex Kinetex column (250 mm × 10.0 mm, 5 μm; ACN-H_2_O, 25:75, 4 mL/min) on a semi-preparative HPLC (waters, 2495 series equipped with a photodiode array detector), which eventually afford the active compound 5 mg of MaiA.

To resolve the chemical structure of MaiA, NMR experiments were carried out on a Bruker Avance III 600 MHz spectrometer with DMSO-*d*_6_ as the solvent and the data were analyzed with Bruker Topspin software. Accurate mass spectra of MaiA was recorded using an Agilent 6545 Q-TOF mass spectrometer coupled to a 1260 HPLC system. Single-crystal data were measured on an Agilent Xcalibur diffractometer with an Atlas (Gemini ultra Cu) detector.

### Minimum Inhibitory Concentration and Drug Combination Studies

Minimum inhibitory concentration (MIC) was determined by broth microdilution according to the Clinical and Laboratory Standards Institute guidelines. Bacteria were cultured in cation-adjusted Mueller Hinton broth at 35 ± 2°C. The MIC was defined as the lowest concentration of antibiotics with no visible growth. In order to determine the effects of combinations of MaiA with antibiotics, combinations of various dilutions of MaiA and a second drug were tested for growth inhibition by microdilution. The synergistic effect was evaluated by the fractional inhibitory concentration (FIC) index (Odds, 2003).

### Anti-biofilm Assays

An anti-biofilm formation assay was performed as previously described with minor modifications (Nair et al., 2016; Park et al., 2016). Briefly, an overnight culture of each strain was diluted in MH medium (for Gram-negative strains), or LB supplemented with 0.5% glucose (for Gram-positive strains) to achieve an OD_595_ value of 0.1. Then incubated statically in a 24-well flat-bottom polystyrene plate (Jet Bio-Filtration, Co., Ltd., China) with or without the varying concentrations of compounds at 37°C for 24 h. Subsequently, planktonic cells were discarded and the wells were rinsed twice with sterile PBS gently and then dried. Biofilms were stained with 0.1% crystal violet (CV) and dissolved in 30% acetic acid, and absorbance at 570 nm (OD_570_) was measured to quantify biofilm biomass. The minimum biofilm inhibitory concentration (MBIC) was defined as the minimum concentration of compounds or antibiotics showing no color development (Nair et al., 2016). The effect of different concentrations of MaiA on the growth of bacteria was monitored by measuring absorbance at 595 nm using a spectrophotometer.

The metabolic activity of biofilm was examined by a metabolic dye reduction assay using MTT [3-(4,5-dimethyl-2-thiazolyl)-2,5-diphenyl-2H-tetrazolium bromide] according to the previous method (Yin et al., 2019). The activated succinate dehydrogenase in the viable cell of biofilm could reduce the yellow tetrazolium salt (MTT) to formazan, and the resulting product forms a blue solution when dissolved in DMSO. The metabolic activity was correlated to the optical density at 570 nm.

Synergistic activity of MaiA combined with antibiotics was measured following anti-biofilm assay.

### Iron Chelating Activity of MaiA

Maipomycin A dissolved in Mili-Q water (Merck) mixed with aqueous FeSO_4_ or FeCl_3_ solution at the molar ratio of 0:1 and 2:1, respectively. The mixture was then subjected to HPLC and HPLC-Q-TOF MS analysis. Pure MaiA was injected as the standard.

### Iron Rescue Experiment

Effect of addition of exogenous iron on the inhibition of *A. baumannii* ATCC 19606 biofilm formation by MaiA was investigated. *A. baumannii* was treated with 52 μM MaiA for 0, 3, 6, and 12 h, respectively, followed by addition of Fe(II) and Fe(III) ions. Biofilm biomass was quantified by CV stain after 24 h incubation in 24-well flat-bottom plate.

### Confocal Laser Scanning Microscopy Analysis of Biofilm Structure

Anti-biofilm formation assay was established on glass coverslips placed in 24-well flat-bottom plates. After removal of supernatant and rinsed twice with sterile PBS, biofilm cells were stained using the BacLight Live/Dead Viability Kit (L7007, Invitrogen, Carlsbad, United States) at 37°C for 15 min in the dark and imaged with laser confocal microscope Leica TCS SP8 (excitation 488 nm, emission 588 nm) with a 20 × objective. Confocal images were processed using LAS X software to reconstruct 3D views of the biofilm. COMSTAT software was used to calculate biomass (μm^3^/μm^2^), mean thickness (μm), and other parameters (Heydorn et al., 2000).

### Data Analysis

Data were presented as the mean value ± standard deviation of three replicates. Comparison of data between treatments and controls were carried out by one-way ANOVA followed by Tukey’s HSD test using SPSS15.0 software. A *P* value less than 0.05 was considered statistically significant.

## Results

### Identification of the Anti-biofilm Compound From *K. phytohabitans* XY-R10

**FIGURE 1:**
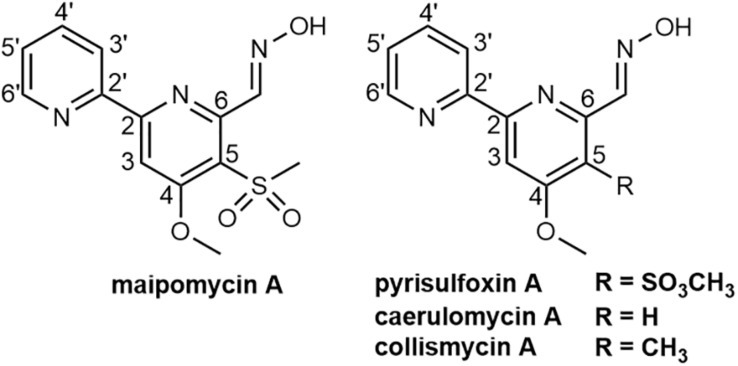
Chemical structures of maipomycin A and its analogs pyrisulfoxin A, caerulomycin A, and collismycin A.

In this study, we found the crude extract of *K. phytohabitans* XY-R10 was able to inhibit the biofilm formation of *A. baumannii* ATCC 19606. Following bioassay-guided fractionation, MaiA with strong anti-biofilm activities was obtained. To identify the chemical structure of MaiA, high-resolution mass, and high-field NMR spectral analyses were carried out (Supplementary Figures 1–7). The ^1^H NMR spectrum of MaiA showed two singlet methyl signals at δ_*H*_ 3.41, 4.16, five doublet olefinic or aromatic signals at δ_*H*_ 7.57, 8.02, 8.20, 8.42, 8.77, 8.84, and one exchangeable proton signal at δ_*H*_ 11.73. The ^13^C NMR and ^1^H NMR data revealed that MaiA contained two methyl groups (δ_*C*_ 44.72, 57.25) and eleven olefinic or aromatic carbon atoms (Supplementary Table 1). These spectroscopic features suggested that MaiA belonged to the family of 2, 2′-bipyridyl, and was most similar to pyrisulfoxin A (PyrA), which was isolated as an antibiotic (Tsuge et al., 1999). The only significant differences in the NMR spectra between these two compounds were the chemical shifts of C-5 (δ_*C*_ 124.5 in 1 vs. δ_*C*_ 127.7 in PyrA) and C-9 (δ_*C*_ 44.72 in 1 vs. δ_*C*_ 39.4 in PyrA). Deduced from the positive ion [M + H]^+^ observed at *m/z* 308.0698 by Q-TOF MS, the molecular formula of MaiA was then determined to be C_13_H_13_N_3_O_4_S, which has one more oxygen atom than that of PyrA (C_13_H_13_N_3_O_3_S). The increase of one oxygen atom, together with the downfield shifts of C-5 and the upfield shifts of C-9, indicated the presence of a sulfonyl group in MaiA, instead of the sulfoxide group in PyrA. HMBC analysis connected the methyl protons H-9 (δ_*H*_ 3.41) to the quaternary carbon C-5 (δ_*C*_ 124.5) of the aromatic ring (Figure 1 and Supplementary Figure 1), for which the methyl sulfonyl group was allocated to the C-5 position. The planar configuration of MaiA was also confirmed by the analysis of the X-ray single-crystal diffraction data (Supplementary Tables 2–8 and Supplementary Figure 8). These data collectively suggested that the anti-biofilm compound isolated from *K. phytohabitans* XY-R10 was 4-methyloxyl-5-methylsulfonyl-2,2′-bipyridyl-6-carboxaldehyde oxime, a novel compound which we named maipomycin A, abbreviated as MaiA.

### MaiA Showed a Weak Antibacterial Activity Against Pathogens

The antibacterial ability of Mai A was evaluated against a panel of Gram-positive and Gram-negative organisms. MIC values indicated that MaiA showed no antibacterial effect against most bacteria (MIC > 256 μg/mL), but had a weak antibacterial activity against *A. baumannii* (MIC = 128 μg/mL) including the reference strains and clinical isolates (Supplementary Table 9).

### MaiA Effectively Inhibit a Variety of GNB Biofilms Formation

**FIGURE 2:**
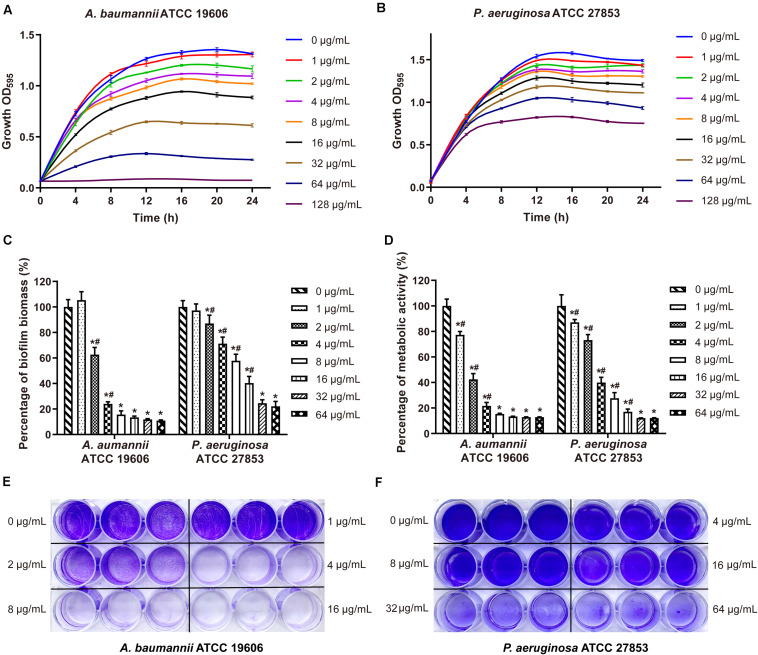
Effects on bacteria growth and biofilm formation by maipomycin A (MaiA). Growth curves of *A. baumannii* **(A)** and *P. aeruginosa* **(B)** treated with various concentrations of MaiA. Effects of MaiA on biomass **(C)** and cell metabolic activity **(D)** in bacterial biofilms. CV stain assay of MaiA on *A. baumannii* **(E)** and *P. aeruginosa* **(F)** biofilm biomass formation in the 24-well flat bottom plate. *Indicates *P* < 0.05 compared with untreated controls. ^#^Indicates *P* < 0.05 compared with the previous concentration.

Maipomycin A was tested in preventing biofilm formation of pathogens using a CV staining method to quantify the production of biofilms biomass. It showed an effective inhibitory activity against the Gram-negative bacteria biofilm formation with a dose-dependent manner from 2 to 64 μg/mL (Figure 2C). The MBIC value of MaiA for *A. baumannii* ATCC 19606 and *P. aeruginosa* ATCC 27853 was 8 and 32 μg/mL, respectively. The percentage of biofilm biomass at MBIC was reduced by 84.3% for *A. baumannii*, and 82.6% for *P. aeruginosa* compared with control. It was notable that MaiA only had a slight inhibition on the planktonic cell growth of these two strains at MBIC concentration (Figures 2A,B), but it could inhibit biofilm formation effectively (Figures 2E,F).

In order to investigate whether MaiA has a broad anti-biofilm formation ability, several strains were selected for further investigation. Results displayed that MaiA could inhibit most Gram-negative bacteria biofilm formation and MBICs ranging from 8 to 32 μg/mL (Supplementary Figures 9A,D–I). However, MaiA had no anti-biofilm activity against *S. aureus* at concentrations up to 64 μg/ml (Supplementary Figures 9B,C).

The metabolic activity of the biofilm cells was measured by MTT staining, which is commonly used for the quantitative analysis of biofilm. The cell metabolic activity of *A. baumannii* and *P. aeruginosa* biofilm decreased significantly and dose-dependently when treated with MaiA (Figure 2D). The MBIC value of MaiA was 8 μg/mL for *A. baumannii*, and 32 μg/mL for *P. aeruginosa* determined by MTT stain. The metabolic activity of both strains was reduced by more than 80% at MBIC.

### Confocal Microscopy Analysis of Biofilm Matrix Upon MaiA Treatment

**FIGURE 3:**
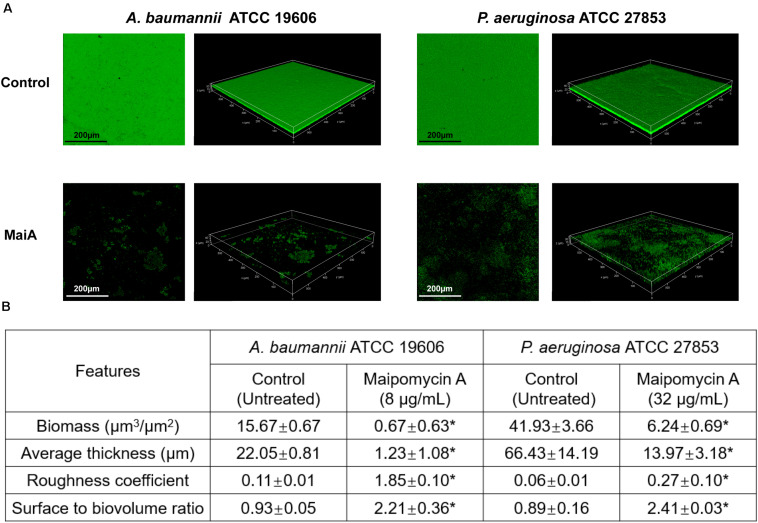
CLSM analyses of biofilm inhibition by maipomycin A (MaiA). **(A)** 3D image of biofilm after 24 h of incubation in glass coverslips. Control was untreated, and MaiA was treated at MBIC concentrations. **(B)** Quantitative analyses of biofilm spatial characteristics using COMSTAT software. The values were expressed as mean ± SD. *Indicates *P* < 0.05 compared with untreated controls.

Confocal laser scanning microscopy (CLSM) was employed to analyze changes in biofilm formation. The reconstructed three-dimensional biofilm images revealed that MaiA at MBIC markedly inhibited biofilm formation compared to control (Figure 3A). The calculation results of COMSTAT software also confirmed the effect of biofilm inhibition. MaiA reduced the biomass (μm^3^/μm^2^) and mean thickness (μm) of *A. baumannii* ATCC 19606 biofilms by >90% and >80% for *P. aeruginosa* ATCC 27853 (Figure 3B). Furthermore, the increases in roughness coefficient and surface to biovolume ratio suggested the heterogeneity and incompleteness of biofilm development. These results also showed that MaiA was an effective agent to inhibit biofilm formation against both *A. baumannii* and *P. aeruginosa*.

### MaiA Is an Iron Chelator and Its Activity Can Be Blocked by Both Fe(II) and Fe(III) Ions

2,2′-Bipyridine-containing compounds such as caerulomycin A (CaeA; Figure 1) exerts its immunosuppressive effect by depleting intracellular irons (Kaur et al., 2015), and ColA (Figure 1) acts as an iron chelator to inhibit tumor cell growth (Kawatani et al., 2016). We, therefore, hypothesized that MaiA might exert its antibiofilm activity through chelating irons, which were essential for both growth and biofilm formation for most bacteria (Schaible and Kaufmann, 2004). In order to prove this hypothesis, we carried out a series of experiments.

**FIGURE 4:**
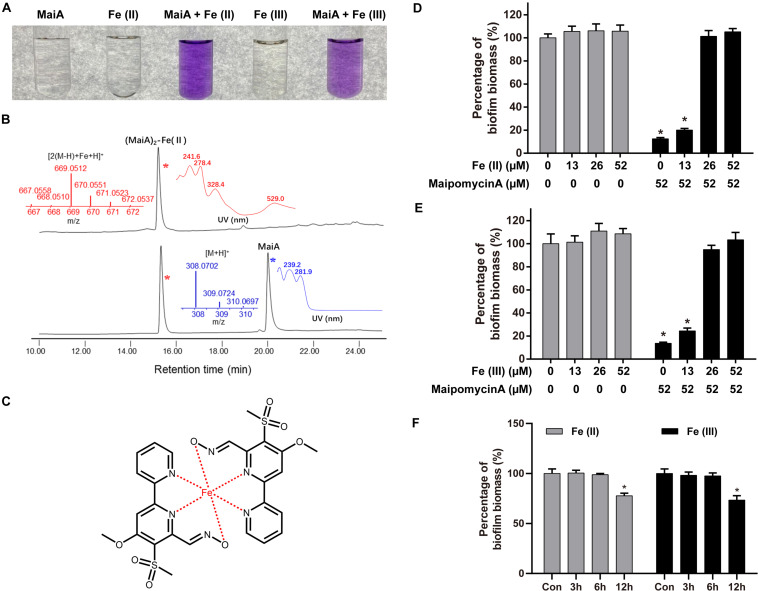
Maipomycin A (MaiA) is an iron chelator. **(A)** Mili-Q water containing MaiA (final concentration at 0.1 mM) treated with Fe(II) or Fe(III) ions (final concentration both at 0.05 mM). **(B)** HPLC analysis (DAD at 244 nm) of the solutions containing MaiA and FeSO_4_ at molar ratios of 2:1. Mass and DAD profiles of MaiA and the (MaiA)_2_-Fe(II) complex are presented in blue and red colors, respectively. **(C)** Putative structure of (MaiA)_2_-Fe complex. **(D,E)** Biofilm formation by *A. baumannii* ATCC 19606 was measured in the presence or absence of iron with (black) or without (gray) the treatment of MaiA. Both Fe(II) and Fe(III) ions were added at the beginning of the incubation. **(F)** MaiA (final concentration at 52 μM) was added to the cultures initially, then Fe(II) or Fe(III; final concentration both at 26 μM) was added after 3, 6, and 12 h, respectively. *Indicates *P* < 0.05 compared with untreated controls (Con).

Firstly, we found that MaiA indeed chelated Fe(II) ions to form a purple complex (Figure 4A). HPLC-MS analysis further confirmed that this complex was formed by two molecules of MaiA and one molecule of Fe (Figures 4B,C). The isotope pattern of this complex in the mass spectrum was also very characteristic for compounds containing an iron atom. The confirmation of MaiA as an iron chelator suggests the bacterium might have originally produced this compound as a siderophore to acquire irons.

Secondly, the addition of exogenous iron together with MaiA could rescue *A. baumannii* ATCC 19606 biofilm formation in a dose-dependent manner (Figures 4D,E). MaiA significantly impaired biofilm formation without the addition of iron. The inhibition was attenuated when the mole ratio of MaiA and iron was 2:1 or 1:1 owing to MaiA completely chelated the additional iron. Moreover, the biofilm biomass did not decrease if Fe(II) was added 3 h or 6 h after treatment of MaiA (Figure 4F). The rescue experiment suggested irons were associated with the MaiA’s anti-biofilm activity.

Maipomycin A could be deactivated regardless of Fe(II) or Fe(III) ions were added. We found that MaiA forms a complex with Fe(III) ions more slowly than Fe(II) ions (Supplementary Figures 10B,C). The color and ultraviolet-visible spectra between (MaiA)_2_-Fe(II) and (MaiA)_2_-Fe(III) are slightly different (Figure 4A and Supplementary Figure 10). Moreover, it seemed difficult to distinguish (MaiA)_2_-Fe(II) from (MaiA)_2_-Fe(III) using HPLC or mass spectrum analysis (Supplementary Figure 13).

### Activities of MaiA Analogs and Synthetic Iron Chelators

**FIGURE 5:**
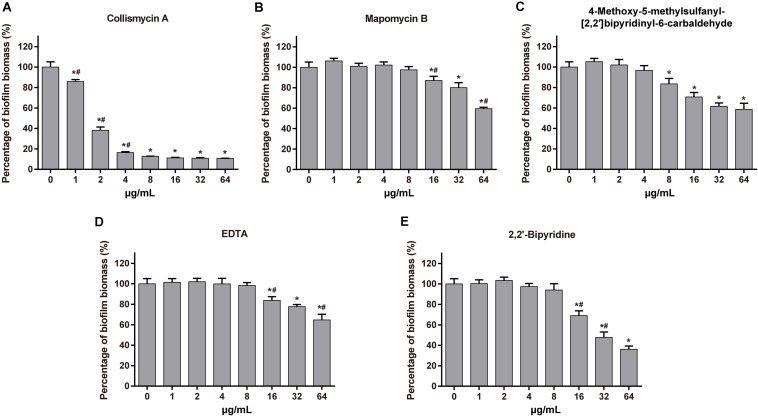
Effect of maipomycin A (MaiA) analogs and synthetic chelators on *A. baumannii* ATCC 19606 biofilm formation. The anti-biofilm activity of tested compounds against *A. baumannii* ATCC 19606 biofilm at different concentrations was quantified by CV stain. The compounds were as follows: **(A)** Collismycin A, **(B)** Maipomycin B, **(C)** 4-Methoxy-5-methylsulfanyl-[2,2′]bipyridinyl-6-carbaldehyde, **(D)** EDTA, and **(E)** 2,2′-dipyridyl. *Indicates *P* < 0.05 compared with untreated controls. ^#^Indicates *P* < 0.05 compared with the previous concentration.

We also investigated the anti-biofilm activity of MaiA’s analogs and other iron chelators. ColA showed strong anti-biofilm activity. The MBIC values of ColA for *A. baumannii* ATCC 19606 and *P. aeruginosa* ATCC 27853 were 8 and 16 μg/mL, respectively, (Figure 5 and Supplementary Figure 11). The activity of the other analogs maipomycin B (MaiB) and 4-Methoxy-5-methylsulfanyl-[2,2′]bipyridinyl-6-carbaldehyde were much weaker than MaiA and ColA. MaiA and ColA both have a 6-carboxaldehyde oxime group, different from the 6-carbaldehyde group of MaiB and 4-Methoxy-5-methylsulfanyl-[2,2′]bipyridinyl-6-carbaldehyde. Therefore, the activity may be related to 6-carboxaldehyde oxime. As two well-known iron chelators, the biofilm inhibition activity of ethylene diamine tetraacetic acid (EDTA) and 2,2′-dipyridyl (2DP) was weaker than MaiA or ColA.

### MaiA Increases the Efficacy of Colistin Against *A. baumannii*

Different classes of antibiotics were combined with MaiA for screen drug interaction against a panel of Gram-positive and Gram-negative organisms. We found that MaiA itself only displayed a weak antibacterial activity but it could potentiate colistin against *A. baumannii* efficiently (Supplementary Table 11). A dose-response study with MaiA was performed against all four *A. baumannii* strains at concentrations of 32, 16, and 8 μg/mL. The MIC of colistin reduced 4–8 folds when used in combination with MaiA, and a synergistic effect between these two compounds could be observed (FICIs ≤ 0.5; Supplementary Table 12). MaiA also enhanced the anti-biofilm activity of colistin yet with only an additive effect. Furthermore, a similar synergistic effect was also observed between colistin and the other two iron chelators ColA and 2DP (Supplementary Table 13).

When MaiA and Fe were treated with a molar ratio of 2:1 after combined with colistin, the enhancement effect completely disappeared (data not shown), indicating that the iron ions might be an interrupter and the MaiA-Fe complex seems not to be the potentiator.

## Discussion

Biofilms are microbial communities of bacteria encased in a self-produced matrix that encrust biotic or abiotic surfaces (Flemming and Wingender, 2010). Biofilm formation is an important virulence factor for a variety of bacteria that cause chronic and persistent infections. Within biofilms, the physiology of bacteria cells would change and become tolerant to antibiotics (Stewart and Costerton, 2001). This causes difficulties in clinical therapeutics and increases the mortality of severe patients. In previous studies, we have reported the butenolide, an antifouling compound derived from a marine *Streptomyces* sp., could effectively inhibit biofilm formation and eradicate preformed biofilm (Yin et al., 2019). In our continuing effort to discover anti-biofilm natural products from marine microbes, a novel secondary metabolite Maipomycin A (which was initially misnamed as Kibdelomycin A; Xu et al., 2017) was isolated from *K. phytohabitans* XY-R10, which showed a promising inhibition of GNB biofilm formation at low concentrations.

Maipomycin A bears a unique 2,2′-bipyridine structure and an unusual oxime functionality. Similar compounds include CaeA (Funk and Divekar, 1959), collismycin A (ColA; Gomi et al., 1994), and pyrisulfoxin A (PyrA; Tsuge et al., 1999). Interestingly, CaeA, ColA, and PyrA were all initially isolated from different *Streptomyces* strains, while MaiA was isolated from *K. phytohabitans*, a non-*Streptomycete* actinomycete. Furthermore, it is remarkable that MaiA contains a sulfone moiety which is extremely rare in natural products with only two known examples, echinosulfone A and sulfadixiamycins (Dunbar et al., 2017).

In this study, we found that MaiA was able to inhibit biofilm formation of GNB, but not effective for gram-positive bacteria (GPB). MaiA showed a limited impact on planktonic growth of the tested GNB strains at MBIC, indicating it did not prevent biofilm development through antibacterial activities. Concerning the rapid development of antibiotic resistance, MaiA could be a safer agent in which poses less selective pressure to bacteria compared to the antibiotics when used for preventing biofilm infections. Furthermore, we also found MaiA inhibits *A. baumannii* from forming biofilms on medical materials *in vitro*, such as catheters (silicone) and endotracheal tubes (polyvinyl chloride; Supplementary Figure 12). These results showed that MaiA may have a good potential as a broad-spectrum anti-GNB biofilm agent in future applications.

Iron is known to play a significant role in bacterial growth and is relevant for biofilm formation (Schaible and Kaufmann, 2004; Banin et al., 2005). Many exogenous iron chelators have been discovered as anti-biofilm agents, such as lactoferrin (Singh et al., 2002), EDTA (Banin et al., 2006), 2,2′-dipyridyl (O’May et al., 2009), and cahuitamycins (Park et al., 2016). Two approved drugs, deferasirox, and deferoxamine as iron scavengers can reduce biofilm formation in cystic fibrosis cells (Moreau-Marquis et al., 2009). Recently, a 2,2′-bipyridine-containing compound collismycin C is reported to inhibit biofilm formation of *S. aureus* (Lee et al., 2017). The anti-biofilm activity was blocked when Fe(III) ions were added together with collismycin C. Similar results were also obtained in our study as both Fe(II) and Fe(III) ions can interrupt the activity of MaiA. The compounds used in this study have different efficacies against biofilms. Among the compounds we tested, only MaiA and ColA showed good activity against biofilms of *A. baumannii* and *P. aeruginosa*. Once EDTA was incubated with MaiA as a competitor, the amount of complex formed by MaiA and iron was greatly reduced (Supplementary Figures 14B,C). The affinity of MaiA toward Fe(II) and Fe(III) ions seem to be weaker than EDTA, but MaiA was much better than EDTA in regard to the anti-biofilm activity. In our exploration of the metal selectivity of MaiA, it may not only chelate Fe(II) and Fe(III) ions. The chelation of MaiA and iron ions may be negatively affected by other metal as competitors (Supplementary Figure 15). Our findings suggested that the ability to chelate iron may not be the sole basis for MaiA activity. The connection among chelators, iron acquisition and biofilm formation in different pathogens requires further investigation.

Caerulomycin A (Funk and Divekar, 1959), ColA (Gomi et al., 1994), and 2DP (Thompson et al., 2012) have been reported to have antibacterial activity. In our results, MaiA or ColA could act as synergists to increase the efficacy of colistin against *A. baumannii*. Colistin is the last-line agent to treat GNB infections, but it is easy to cause neurotoxicity and nephrotoxicity because of the narrow therapeutic indices (Falagas and Kasiakou, 2006). One promising strategy to overcome the toxicity of colistin is to combine its use with synergists to enhance the antibacterial effect without increasing the dose of drug (Lenhard et al., 2016). Therefore, MaiA and ColA may be able to provide potential solutions. Another iron chelator *N*,*N*′-bis (2-hydroxybenzyl) ethylenediamine-*N*,*N*′-diacetic acid combined with colistin is highly effective against biofilms of *P. aeruginosa* (Mettrick et al., 2020). Slight enhancements of anti-biofilm were observed when colistin coupled with MaiA, ColA, or 2DP. The mechanistic investigation of how iron chelators increase the antibacterial activity of colistin is currently underway.

In summary, we have discovered a novel compound Maipomycin A from *K. phytohabitans* XY-R10 and it could effectively inhibit biofilm formation of most Gram-negative bacteria with a minor antibacterial effect. MaiA turned out to be an iron chelator and its antibiofilm activity could be blocked by both Fe(II) and Fe(III) ions. In addition, MaiA showed the effect of enhancing colistin against *A. baumannii*. These combined results suggest MaiA might be a promising candidate for the development of a new anti-biofilm agent and colistin enhancer with great potential in the future application.

## Data Availability Statement

The raw data supporting the conclusions of this article will be made available by the authors, without undue reservation.

## Author Contributions

YX, CW, and JZ planned experiments. JZ, XL, SZ, and ZS conducted experiments. The manuscript was written by YX and JZ. All authors read and approved the manuscript prior to submission.

## Conflict of Interest

The authors declare that the research was conducted in the absence of any commercial or financial relationships that could be construed as a potential conflict of interest.
