# Supplementary material for: Maipomycin A, a Novel Natural Compound With Promising Anti-biofilm Activity Against Gram-Negative Pathogenic Bacteria
Source: Front Microbiol. 2021 Jan 12;11:598024. doi: 10.3389/fmicb.2020.598024 (PMC7835661; doi:10.3389/fmicb.2020.598024)
Supplement: Supplementary file 1 [file Data_Sheet_1.docx]

**Supplementary Information**

**Maipomycin A, a novel natural compound with promising anti-biofilm activity against Gram-negative pathogenic bacteria**


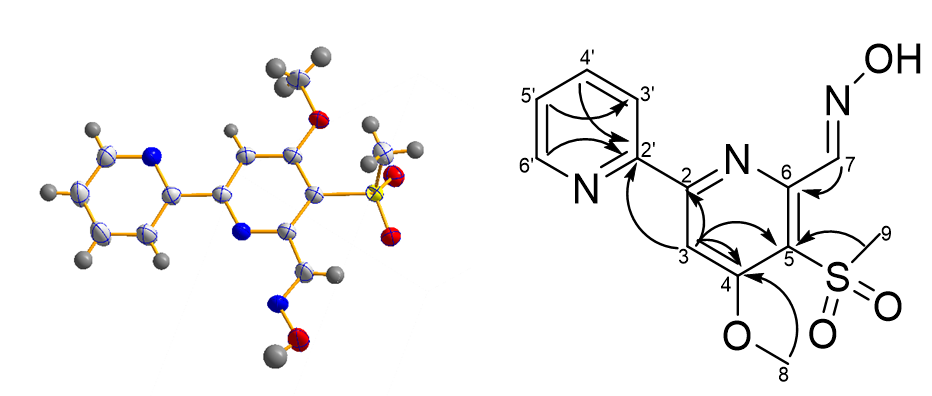


**Supplementary Figure 1.** X-ray crystal structure of maipomycin A and key HMBC correlations in maipomycin A.

**Supplementary Figure 2.** ^1^H NMR (600 MHz, *d*_6_-DMSO) spectrum of maipomycin A.

**Supplementary Figure 3.** ^13^C NMR (600 MHz, *d*_6_-DMSO) spectrum of maipomycin A.


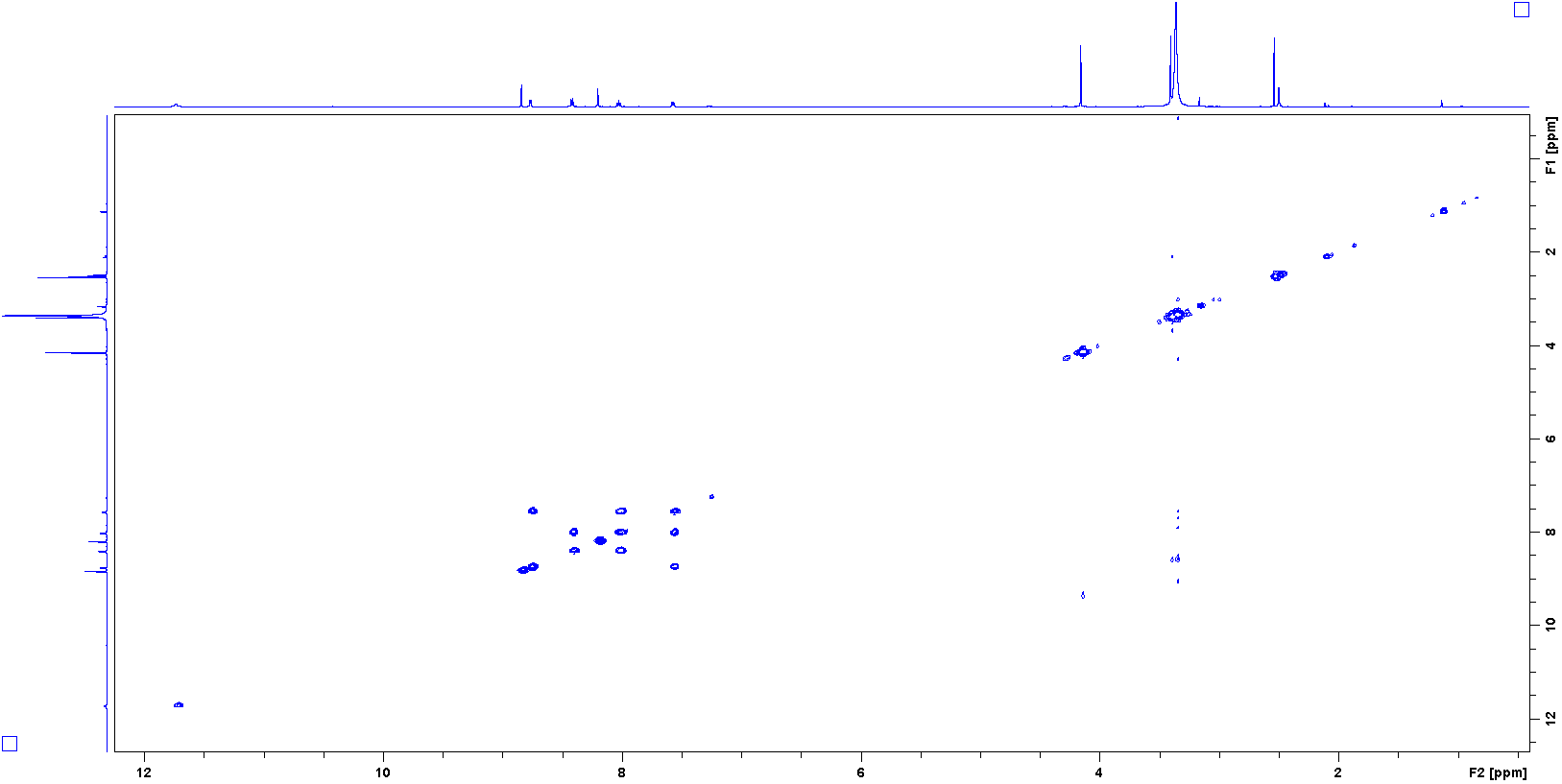


**Supplementary Figure 4.** ^1^H–^1^H COSY **(***d*_6_-DMSO) spectrum of maipomycin A.

**Supplementary Figure 5.** HSQC **(***d*_6_-DMSO) spectrum of maipomycin A.

**Supplementary Figure 6.** HMBC **(***d*_6_-DMSO) spectrum of maipomycin A.


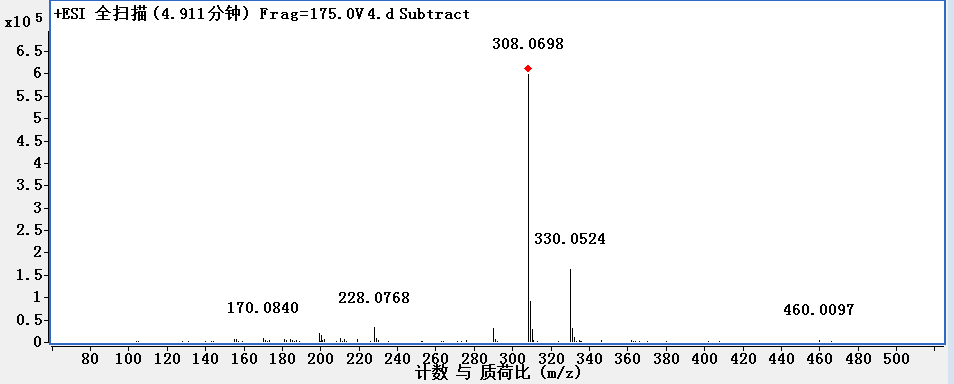


**Supplementary Figure 7**. HR-ESI-MS spectrum of maipomycin A.


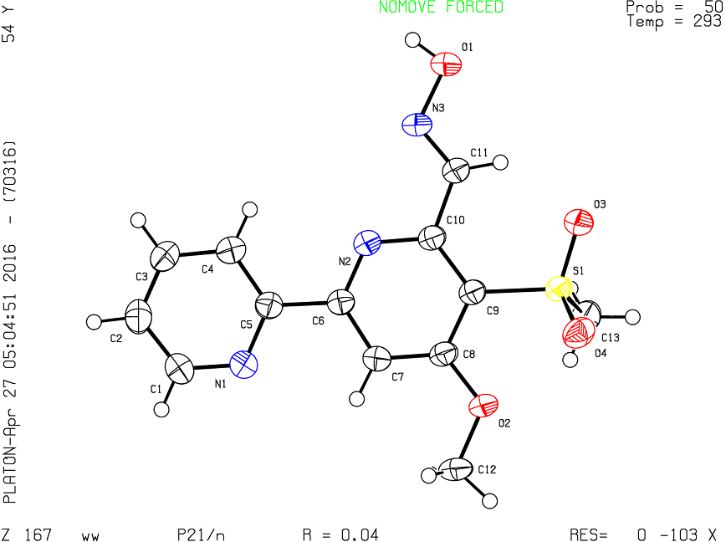


**Supplementary Figure 8.** Crystal structure determination of maipomycin A. Crystal data. C_13_H_13_N_3_O_4_S, *M*=307.32，monoclinic, *a* = 12.6716(4) Å, b = 6.8261(2) Å, c = 16.3151(4) Å, *β* = 100.612(3)°, *U*=1387.08(7) Å^3^，*T*=293.2, space group P 2_1_/n (no. 14), Z=4, μ (Cu Kα) = 0.856, 156654 reflections collected, 2409 unique (*R*_int_ = 0.0704) which were used in all calculations. The final *wR* (*F*_2_) was 0.1176 (all data).


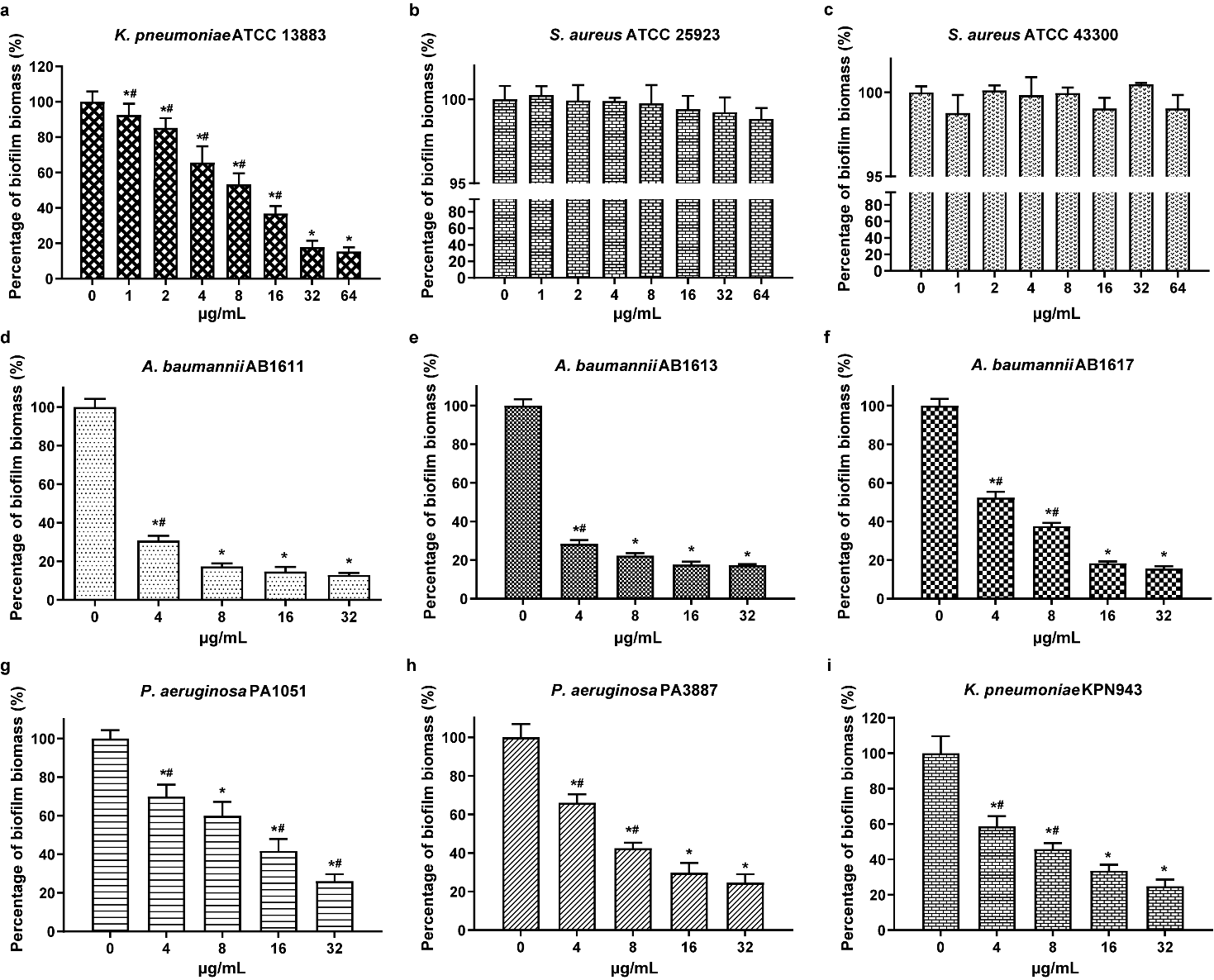


**Supplementary Figure 9.** Inhibitory activity of maipomycin A on biofilm formation in other Gram-positive and Gram-negative strains. (a) to (c) were reference strains, and (d) to (i) were clinical isolates. * indicates *P* < 0.05 compared with untreated controls. # indicates *P* < 0.05 compared with the previous concentration.


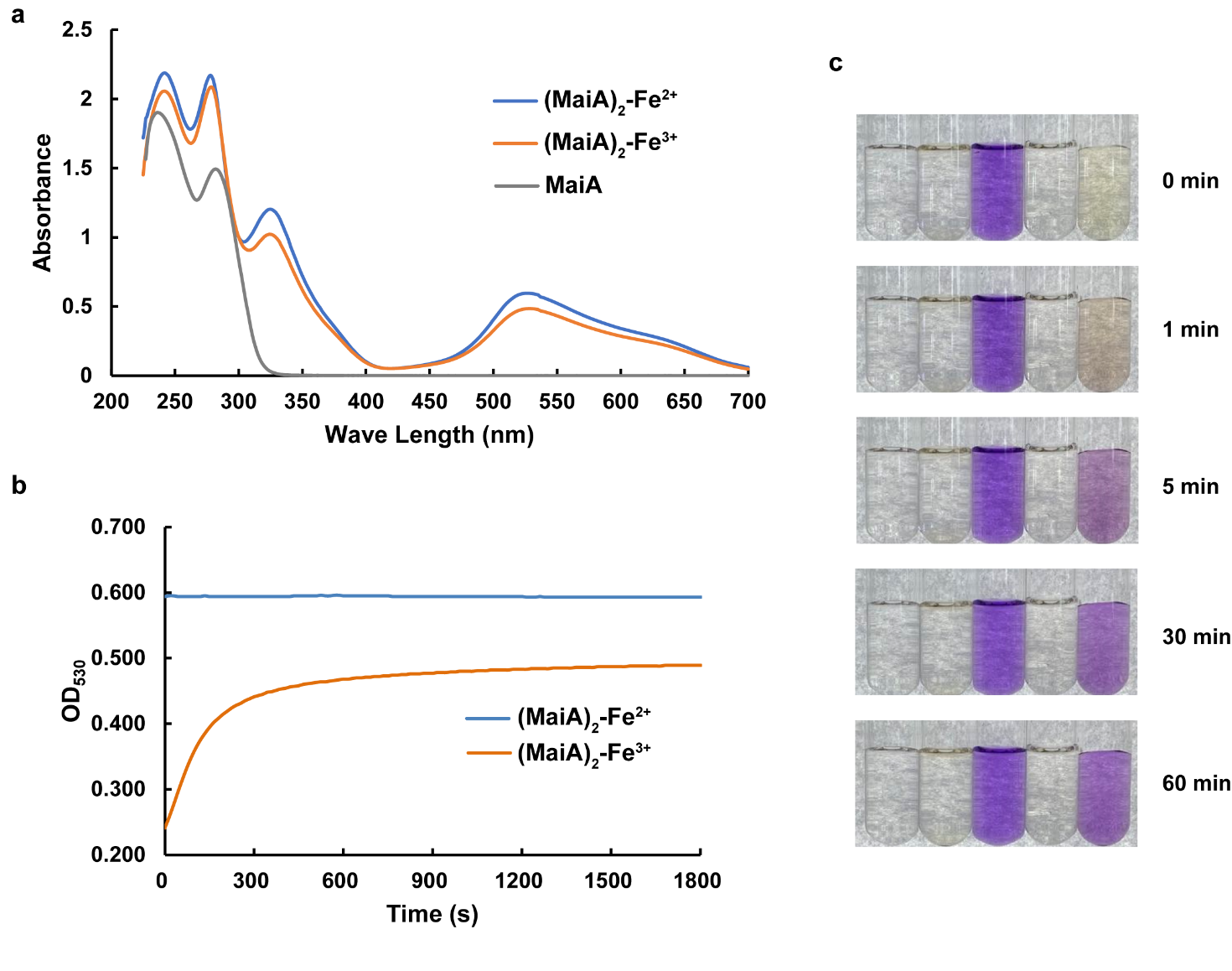


**Supplementary Figure 10.** Maipomycin A chelate Fe(Ⅱ) or Fe(Ⅲ) ions showed different properties. (a) Ultraviolet-visible spectra of MaiA (0.1mM), (MaiA)_2_-Fe(Ⅱ) (0.05mM) and (MaiA)_2_-Fe(Ⅲ) (0.05mM). (b) Determination of the time for coordination reaction of (MaiA)_2_-Fe(Ⅱ) and (MaiA)_2_-Fe(Ⅲ) to reach equilibrium. (c) MaiA chelate Fe(Ⅲ) showed different colors before reaching equilibrium. The samples in the tube are MaiA, Fe(Ⅱ), (MaiA)_2_-Fe(Ⅱ), Fe(Ⅲ), (MaiA)_2_-Fe(Ⅲ) from left to right.


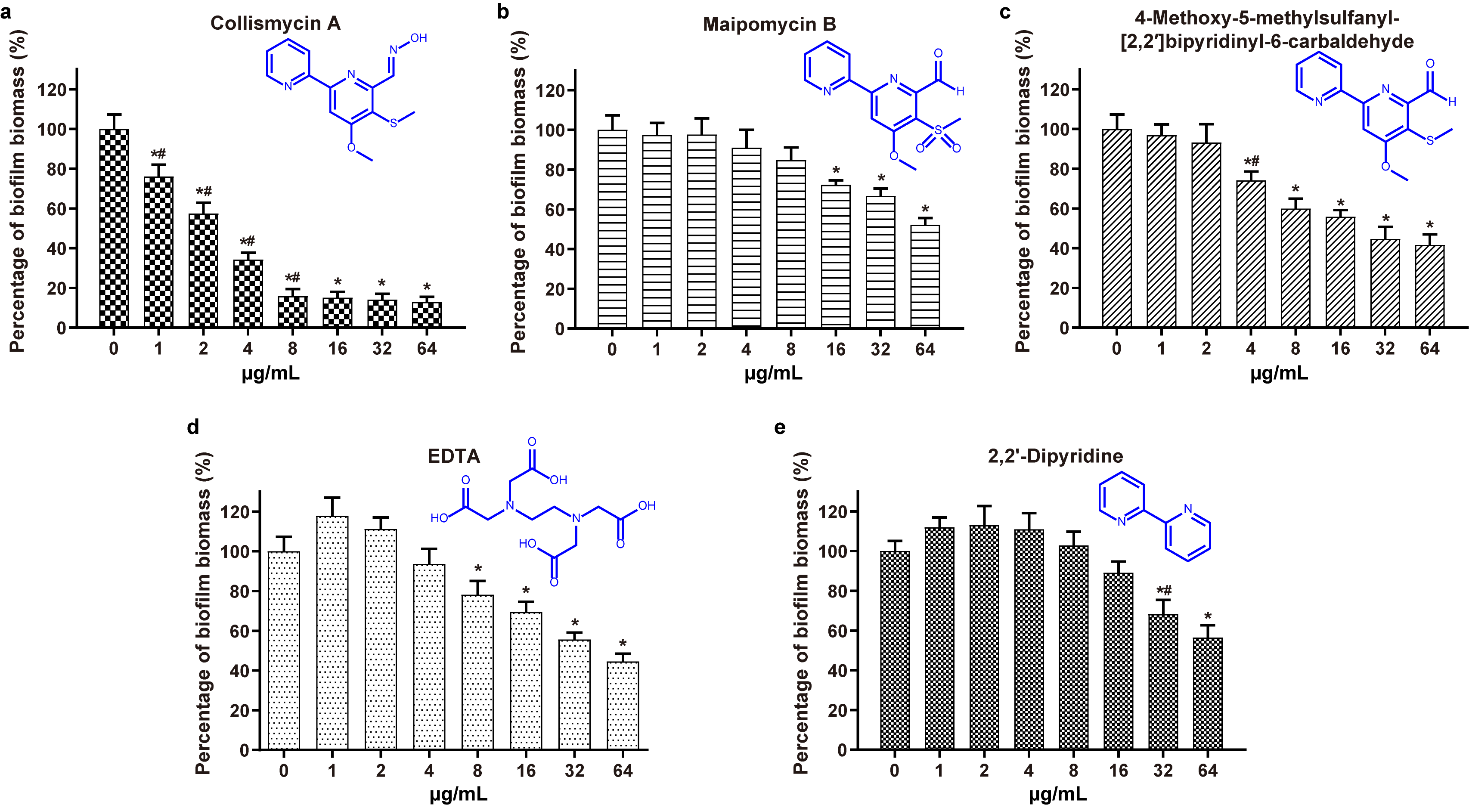


**Supplementary Figure 11.** Effect of synthetic chelators and analogs of maipomycin A on *P. aeruginosa* ATCC 27853 biofilm formation. The anti-biofilm activity of tested compounds against *P. aeruginosa* ATCC 27853 biofilm at different concentrations was quantified by CV stain. The structure of each compound is shown in the chart. * indicates *P* < 0.05 compared with untreated controls. # indicates *P* < 0.05 compared with the previous concentration.


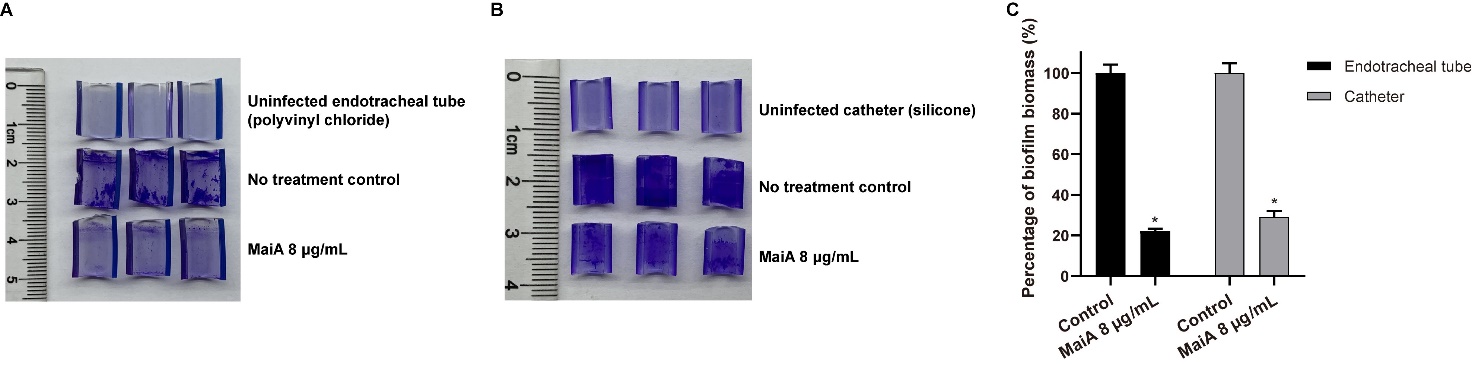


**Supplementary Figure 12.** The activity of maipomycin A against *A. baumannii* ATCC 19606 biofilms on endotracheal tubes and catheters. Endotracheal tubes and catheters pieces of 1 to 2 cm were cut, sliced into two halves, added to the culture and incubated with *A. baumannii.* Then stained (A, B) and quantified (C) by CV assay.

**Supplementary Table 1.** ^1^NMR Data (600 MHz, *δ* in ppm, *J* in Hz) and ^13^C NMR Data (150 MHz, *δ* in ppm) for maipomycin A (MaiA) and pyrisulfoxin A (PyrA).

|  | | **MaiA***^a^* | |  | | | **PyrA***^b^* | | |
| --- | --- | --- | --- | --- | --- | --- | --- | --- | --- |
| position | *δ*_C_, type | | *δ*_H_, mult  (*J* in Hz) | |  | *δ*_C_, type | | *δ*_H_, mult (*J* in Hz) |  |
| 2 | 159.4, C | |  | |  | 159.7, C | |  |  |
| 3 | 104.4, CH | | 8.20, s | |  | 104.3, CH | | 8.12,s |  |
| 4 | 165.3, C | |  | |  | 165.6, C | |  |  |
| 5 | 124.5, C | |  | |  | 127.7, C | |  |  |
| 6 | 150.4, C | |  | |  | 150.6, C | |  |  |
| 7 | 147.3, CH | | 8.84, s | |  | 147.7, CH | | 8.88,s |  |
| 8 | 57.2, CH_3_ | | 4.16, s | |  | 56.6, CH3 | | 4.13,s |  |
| 9 | 44.7, CH_3_ | | 3.41, s | |  | 39.4, CH3 | | 3.06,s |  |
| 2’ | 153.4, C | |  | |  | 154.4, C | |  |  |
| 3’ | 121.8, CH | | 8.42, d  (7.98) | |  | 122.0, CH | | 8.50, ddd  (8.0, 1.3, 1.0) |  |
| 4’ | 137.2, CH | | 8.02, ddd  (7.68, 7.5, 1.56) | |  | 137.2, CH | | 7.83, ddd  (8.0, 7.5, 1.8) |  |
| 5’ | 126.5, CH | | 7.57, m | |  | 124.9, CH | | 7.39, ddd  (7.5, 4.9, 1.1) |  |
| 6’ | 150.6, CH | | 8.77, d  (4.2) | |  | 149.1, CH | | 8.68, ddd  (4.8, 1.8, 1.0) |  |
| N-OH |  | | 11.73, s | |  |  | |  |  |

*^a^*DMSO-*d*_6_. *^b^*CDCl_3_. *^b^*Data were reported by Tsuge et al.

**Supplementary Table 2.** Crystal data and structure refinement for maipomycin A (MaiA)

| Identification code | MaiA |
| --- | --- |
| Empirical formula | C_13_H_13_N_3_O_4_S |
| Formula weight | 307.32 |
| Temperature / K | 293.2 |
| Crystal system | monoclinic |
| Space group | P 2_1_/ n |
| a / Å, b / Å, c / Å | 12.6716(4), 6.8261(2), 16.3151(4) |
| α/°, β/°, γ/° | 90.00, 100.612(3), 90.00 |
| Volume / Å^3^ | 1387.08(7) |
| Z | 4 |
| ρ_calc_ / mg mm^-3^ | 1.472 |
| μ / mm^‑1^ | 2.273 |
| F(000) | 640 |
| Crystal size / mm^3^ | 0.21 × 0.20× 0.19 |
| 2Θ range for data collection | 4.07 to 66.07 |
| Index ranges | -15 ≤ h ≤ 14, -7 ≤ k ≤ 8, -19 ≤ l ≤ 18 |
| Reflections collected | 15654 |
| Independent reflections | 2409[R(int) = 0.0704 (inf-0.9Å)] |
| Data/restraints/parameters | 2409/1/191 |
| Goodness-of-fit on F^2^ | 1.047 |
| Final R indexes [I>2σ (I) i.e. F_o_>4σ (F_o_)] | R_1_ = 0.0410, wR_2_ = 0.1111 |
| Final R indexes [all data] | R_1_ = 0.0462, wR_2_ = 0.1176 |
| Largest diff. peak/hole / e Å^-3^ | 0.195/-0.484 |
| Completeness | 0.998 |

**Supplementary Table 3.** Fractional Atomic Coordinates (×10^4^) and Equivalent Isotropic Displacement Parameters (Å^2^×10^3^) for maipomycin A. U_eq_ is defined as 1/3 of the trace of the orthogonalized U_ij_ tensor

| **Atom** | ***x*** | ***y*** | ***z*** | **U(eq)** |
| --- | --- | --- | --- | --- |
| S1 | 3187(1) | 2025(1) | 1487(1) | 34(1) |
| O1 | 4178(2) | 1821(3) | 4729(1) | 86(1) |
| O2 | 3781(1) | -1188(2) | 524(1) | 45(1) |
| O3 | 3103(1) | 3372(2) | 2148(1) | 49(1) |
| O4 | 3584(1) | 2792(2) | 785(1) | 44(1) |
| N1 | 6047(1) | -6287(2) | 2267(1) | 40(1) |
| N2 | 5021(1) | -1870(2) | 3029(1) | 35(1) |
| N3 | 4522(1) | 568(3) | 4160(1) | 45(1) |
| C1 | 6641(2) | -7835(3) | 2558(1) | 47(1) |
| C2 | 7140(2) | -8052(3) | 3371(1) | 54(1) |
| C3 | 7019(2) | -6593(4) | 3924(1) | 62(1) |
| C4 | 6386(2) | -5000(3) | 3651(1) | 52(1) |
| C5 | 5914(1) | -4891(3) | 2816(1) | 35(1) |
| C6 | 5242(1) | -3180(3) | 2474(1) | 32(1) |
| C7 | 4858(2) | -3034(3) | 1626(1) | 36(1) |
| C8 | 4223(1) | -1444(3) | 1331(1) | 34(1) |
| C9 | 3998(1) | -21 (2) | 1899(1) | 33(1) |
| C10 | 4396(1) | -329 (3) | 2750(1) | 35(1) |
| C11 | 4130(2) | 930 (4) | 3420(1) | 60(1) |
| C12 | 4010(2) | -2586(4) | -76(1) | 61(1) |
| C13 | 1917(2) | 966(3) | 1147(1) | 44(1) |

**Supplementary Table 4.** Anisotropic Displacement Parameters (Å^2^×10^3^) for maipomycin A. The Anisotropic displacement factor exponent takes the form: -2π^2^ [h^2^a*^2^U_11_+...+2hka×b×U_12_].

| Atom | U11 | U22 | U33 | U23 | U13 | U12 |
| --- | --- | --- | --- | --- | --- | --- |
| S1 | 44(1) | 31(1) | 25(1) | 1(1) | 3(1) | 3(1) |
| O1 | 142(2) | 82(1) | 28(1) | -11(1) | 1(1) | 66(1) |
| O2 | 65(1) | 46(1) | 22(1) | -4(1) | 2(1) | 15(1) |
| O3 | 72(1) | 40(1) | 33(1) | -6(1) | 1(1) | 16(1) |
| O4 | 56(1) | 40(1) | 35(1) | 7(1) | 6(1) | -5(1) |
| N1 | 45(1) | 40(1) | 35(1) | -3(1) | 5(1) | 8(1) |
| N2 | 41(1) | 36(1) | 27(1) | 0(1) | 4(1) | 4(1) |
| N3 | 62(1) | 45(1) | 25(1) | -8(1) | 3(1) | 13(1) |
| C1 | 52(1) | 43(1) | 45(1) | -2(1) | 10(1) | 13(1) |
| C2 | 59(1) | 56(1) | 47(1) | 10(1) | 10(1) | 25(1) |
| C3 | 76(2) | 75(2) | 33(1) | 4(1) | 3(1) | 35(1) |
| C4 | 66(1) | 58(1) | 32(1) | -3(1) | 4(1) | 22(1) |
| C5 | 35(1) | 37(1) | 32(1) | 0(1) | 7(1) | 2(1) |
| C6 | 33(1) | 34(1) | 29(1) | -1(1) | 7(1) | 0(1) |
| C7 | 43(1) | 36(1) | 29(1) | -3(1) | 7(1) | 4(1) |
| C8 | 41(1) | 37(1) | 24(1) | 0(1) | 5(1) | 0(1) |
| C9 | 38(1) | 32(1) | 26(1) | 0(1) | 4(1) | 1(1) |
| C10 | 43(1) | 34(1) | 26(1) | -1(1) | 3(1) | 2(1) |
| C11 | 97(2) | 52(1) | 27(1) | -2(1) | 0(1) | 35(1) |
| C12 | 99(2) | 58(1) | 24(1) | -7(1) | 7(1) | 22(1) |
| C13 | 42(1) | 47(1) | 41(1) | 6(1) | 6(1) | 3 (1) |

**Supplementary Table 5.** Bond Lengths for maipomycin A

| Atom | Atom | Length/Å |  | Atom | Atom | Length/Å |
| --- | --- | --- | --- | --- | --- | --- |
| S1 | O4 | 1.4323(14) |  | C3 | C4 | 1.376(3) |
| S1 | O3 | 1.4352(13) |  | C3 | H3A | 0.9300 |
| S1 | C13 | 1.7580(19) |  | C4 | C5 | 1.386(3) |
| S1 | C9 | 1.7894(17) |  | C4 | H4A | 0.9300 |
| O1 | N3 | 1.391(2) |  | C5 | C6 | 1.492(3) |
| O1 | H1D | 0.8607 |  | C6 | C7 | 1.383(3) |
| O2 | C8 | 1.344(2) |  | C7 | C8 | 1.383(3) |
| O2 | C12 | 1.434(2) |  | C7 | H7A | 0.9300 |
| N1 | C1 | 1.333(3) |  | C8 | C9 | 1.408(2) |
| N1 | C5 | 1.339(2) |  | C9 | C10 | 1.403(2) |
| N2 | C6 | 1.338(2) |  | C10 | C11 | 1.477(3) |
| N2 | C10 | 1.345(2) |  | C11 | H11A | 0.9300 |
| N3 | C11 | 1.243(3) |  | C12 | H12A | 0.9600 |
| C1 | C2 | 1.369(3) |  | C12 | H12B | 0.9600 |
| C1 | H1A | 0.9300 |  | C12 | H12C | 0.9600 |
| C2 | C3 | 1.371(3) |  | C13 | H13A | 0.9600 |
| C2 | H2A | 0.9300 |  | C13 | H13B | 0.9600 |
|  |  |  |  | C13 | H13C | 0.9600 |

**Supplementary Table 6.** Bond Angles for maipomycin A

| **Atom** | **Atom** | **Atom** | **Angle/˚** |  | **Atom** | **Atom** | **Atom** | **Angle/˚** |
| --- | --- | --- | --- | --- | --- | --- | --- | --- |
| O4 | S1 | O3 | 116.83(9) |  | C7 | C6 | C5 | 120.12(15) |
| O4 | S1 | C13 | 109.47(8) |  | C6 | C7 | C8 | 118.71(16) |
| O3 | S1 | C13 | 108.09(10) |  | C6 | C7 | H7A | 120.6 |
| O4 | S1 | C9 | 108.90(8) |  | C8 | C7 | H7A | 120.6 |
| O3 | S1 | C9 | 109.57(8) |  | O2 | C8 | C7 | 123.67(16) |
| C13 | S1 | C9 | 103.09(9) |  | O2 | C8 | C9 | 117.20(15) |
| N3 | O1 | H1D | 101.0 |  | C7 | C8 | C9 | 119.12(15) |
| C8 | O2 | C12 | 118.79(15) |  | C10 | C9 | C8 | 117.96(15) |
| C1 | N1 | C5 | 117.28(15) |  | C10 | C9 | S1 | 124.43(13) |
| C6 | N2 | C10 | 118.55(14) |  | C8 | C9 | S1 | 117.58(12) |
| C11 | N3 | O1 | 114.09(17) |  | N2 | C10 | C9 | 122.26(16) |
| N1 | C1 | C2 | 124.11(19) |  | N2 | C10 | C11 | 113.93(14) |
| N1 | C1 | H1A | 117.9 |  | C9 | C10 | C11 | 123.75(18) |
| C2 | C1 | H1A | 117.9 |  | N3 | C11 | C10 | 119.91(18) |
| C1 | C2 | C3 | 118.13(18) |  | N3 | C11 | H11A | 120.0 |
| C1 | C2 | H2A | 120.9 |  | C10 | C11 | H11A | 120.0 |
| C3 | C2 | H2A | 120.9 |  | O2 | C12 | H12A | 109.5 |
| C2 | C3 | C4 | 119.39(18) |  | O2 | C12 | H12B | 109.5 |
| C2 | C3 | H3A | 120.3 |  | H12A | C12 | H12B | 109.5 |
| C4 | C3 | H3A | 120.3 |  | O2 | C12 | H12C | 109.5 |
| C3 | C4 | C5 | 118.71(19) |  | H12A | C12 | H12C | 109.5 |
| C3 | C4 | H4A | 120.6 |  | H12B | C12 | H12C | 109.5 |
| C5 | C4 | H4A | 120.6 |  | S1 | C13 | H13A | 109.5 |
| N1 | C5 | C4 | 122.34(16) |  | S1 | C13 | H13B | 109.5 |
| N1 | C5 | C6 | 116.14(14) |  | H13A | C13 | H13B | 109.5 |
| C4 | C5 | C6 | 121.51(16) |  | S1 | C13 | H13C | 109.5 |
| N2 | C6 | C7 | 123.32(15) |  | H13A | C13 | H13C | 109.5 |
| N2 | C6 | C5 | 116.53(14) | N2 | H13B | C13 | H13C | 109.5 |

**Supplementary Table 7.** Torsion Angles for maipomycin A

| **A** | **B** | **C** | **D** | **Angle/˚** |
| --- | --- | --- | --- | --- |
| C5 | N1 | C1 | C2 | -1.7(3) |
| N1 | C1 | C2 | C3 | 0.2(4) |
| C1 | C2 | C3 | C4 | 1.7(4) |
| C2 | C3 | C4 | C5 | -2.0(4) |
| C1 | N1 | C5 | C4 | 1.3(3) |
| C1 | N1 | C5 | C6 | -179.77(17) |
| C3 | C4 | C5 | N1 | 0.5(3) |
| C3 | C4 | C5 | C6 | -178.4(2) |
| C10 | N2 | C6 | C7 | 0.0(3) |
| C10 | N2 | C6 | C5 | -178.36(15) |
| N1 | C5 | C6 | N2 | 1773.94(15) |
| C4 | C5 | C6 | N2 | -7.1(3) |
| N1 | C5 | C6 | C7 | -4.5(2) |
| C4 | C5 | C6 | C7 | 174.48(19) |
| N2 | C6 | C7 | C8 | 0.7(3) |
| C5 | C6 | C7 | C8 | 179.04(16) |
| N2 | C6 | C7 | C8 | 0.7(3) |
| C5 | C6 | C7 | C8 | 179.04(16) |
| C12 | O2 | C8 | C7 | -2.7(3) |
| C12 | O2 | C8 | C9 | 178.76(19) |
| C6 | C7 | C8 | O2 | -177.88(17) |
| C6 | C7 | C8 | C9 | 0.6(3) |
| O2 | C8 | C9 | C10 | 176.02(16) |
| C7 | C8 | C9 | C10 | -2.6(3) |
| O2 | C8 | 9 | S1 | -2.1(2) |
| C7 | C8 | C9 | S1 | 179.25(14) |
| O4 | S1 | C9 | C10 | 134.62(16) |
| O3 | S1 | C9 | C10 | 5.71(19) |
| C13 | S1 | C9 | C10 | -109.19(17) |
| O4 | S1 | C9 | C8 | -47.35(16) |
| O3 | S1 | C9 | C8 | -176.25(14) |
| C13 | S1 | C9 | C8 | 68.85(16) |
| C6 | N2 | C10 | C9 | -2.1(3) |
| C6 | N2 | C10 | C11 | 175.12(19) |
| C8 | C9 | C10 | N2 | 3.4(3) |
| S1 | C9 | C10 | N2 | -178.55(13)- |
| C8 | C9 | C10 | C11 | -173.55(19) |
| S1 | C9 | C10 | C11 | 4.5(3) |
| O1 | N3 | C11 | C10 | -177.8(2) |
| N2 | C10 | C11 | N3 | 3.3(3) |
| C9 | C10 | C11 | N3 | -179.5(2) |

**Supplementary Table 8.** Hydrogen Atom Coordinates (Å×104) and Isotropic Displacement Parameters (Å2×103) for maipomycin A.

| **Atom** | ***x*** | ***y*** | ***z*** | **U(eq)** |
| --- | --- | --- | --- | --- |
| S1 | 3187(1) | 2025(1) | 1487(1) | 34(1) |
| O1 | 4178(2) | 1821(3) | 4729(1) | 86(1) |
| O2 | 3781(1) | -1188(2) | 524(1) | 45(1) |
| O3 | 3103(1) | 3372(2) | 2148(1) | 49(1) |
| O4 | 3584(1) | 2792(2) | 785(1) | 44(1) |
| N1 | 6047(1) | -6287(2) | 2267(1) | 40(1) |
| N2 | 5021(1) | -1870(2) | 3029(1) | 35(1) |
| N3 | 4522(1) | 568(3) | 4160(1) | 45(1) |
| C1 | 6641(2) | -7835(3) | 2558(1) | 47(1) |
| C2 | 7140(2) | -8052(3) | 3371(1) | 54(1) |
| C3 | 7019(2) | -6593(4) | 3924(1) | 62(1) |
| C4 | 6386(2) | -5000(3) | 3651(1) | 52(1) |
| C5 | 5914(1) | -4891(3) | 2816(1) | 35(1) |
| C6 | 5242(1) | -3180(3) | 2474(1) | 32(1) |
| C7 | 4858(2) | -3034(3) | 1626(1) | 36(1) |
| C8 | 4223(1) | -1444(3) | 1331(1) | 34(1) |
| C9 | 3998(1) | -21(2) | 1899(1) | 33(1) |
| C10 | 4396(1) | -329(3) | 2750(1) | 35(1) |
| C11 | 4130(2) | 930(4) | 3420(1) | 60(1) |
| C12 | 4010(2) | -2586(4) | -76(1) | 61(1) |
| C13 | 117(2) | 966(3) | 1147(1) | 44(1) |

**Supplementary Table 9.** MIC values for maipomycin A (MaiA) and other compounds against a panel of Gram-positive and Gram-negative pathogens.

| Organism | | | MaiA | ColA | MaiB | ColX | 2DP | EDTA | TOB | CLS | VAN | OXA |
| --- | --- | --- | --- | --- | --- | --- | --- | --- | --- | --- | --- | --- |
| Gram- | *A. baumannii* | ATCC 19606 | 128 | 64 | 256 | >256 | 64 | >256 | 2 | 2 | - | - |
|  |  | AB1611* | 128 | 64 | 256 | >256 | 64 | >256 | >32 | 1 | - | - |
|  |  | AB1613* | 128 | 64 | 256 | >256 | 64 | >256 | >32 | 1 | - | - |
|  |  | AB1617* | 128 | 64 | 256 | >256 | 64 | >256 | >32 | 2 | - | - |
|  | *P. aeruginosa* | ATCC 27853 | >256 | >256 | >256 | >256 | >256 | >256 | 2 | 1 | - | - |
|  |  | PA1051* | >256 | >256 | >256 | >256 | >256 | >256 | >16 | 2 | - | - |
|  |  | PA3883* | >256 | >256 | >256 | >256 | >256 | >256 | >16 | 1 | - | - |
|  | *K. pneumoniae* | ATCC 13883 | >256 | >256 | >256 | >256 | >256 | >256 | 1 | 2 | - | - |
|  |  | KPN943* | >256 | >256 | >256 | >256 | >256 | >256 | >32 | 2 | - | - |
|  | *E. coli* | ATCC 25922 | >256 | >256 | >256 | >256 | >256 | >256 | 0.5 | 2 | - | - |
| Gram+ | *S. aureus* | ATCC 25923 (MSSA) | >256 | >256 | >256 | >256 | >256 | >256 | 0.5 | - | 1 | 0.5 |
|  |  | ATCC 43300 (MRSA) | >256 | >256 | >256 | >256 | >256 | >256 | >32 | - | 1 | >64 |

*: Clinical isolates. -: Not tested. MaiA: maipomycin A; ColA: collismycin A; MaiB: maipomycin B; ColX: 4-Methoxy-5-methylsulfanyl-[2,2′]bipyridinyl-6-carbaldehyde; 2DP: 2,2′-dipyridyl; TOB: tobramycin; CLS: colistin; VAN: vancomycin; OXA: oxacillin.

**Supplementary Table 10.** Maipomycin A increase colistin efficacy against *A. baumannii*

| Organism | MIC (μg/mL)  in combination | |  | MBIC (μg/mL)  in combination | |  | MBIC (μg/mL) alone | |
| --- | --- | --- | --- | --- | --- | --- | --- | --- |
|  | MaiA | CLS | FICI | MaiA | CLS | FICI | MaiA | CLS |
| *A. baumannii* ATCC 19606 | 32 | 0.25 | 0.375 | 4 | 0.25 | 0.625 | 8 | 2 |
|  | 16 | 0.5 | 0.375 | 2 | 1 | 0.75 |  |  |
|  | 8 | 1 | 0.5625 | 1 | 2 | 1.125 |  |  |
| *A. baumannii* AB1611* | 32 | 0.125 | 0.375 | 4 | 0.25 | 0.75 | 8 | 1 |
|  | 16 | 0.25 | 0.375 | 2 | 0.5 | 0.75 |  |  |
|  | 8 | 1 | 1.0625 | 1 | 2 | 2.125 |  |  |
| *A. baumannii* AB1613* | 32 | 0.125 | 0.375 | 4 | 0.25 | 0.625 | 8 | 2 |
|  | 16 | 0.25 | 0.375 | 2 | 1 | 0.75 |  |  |
|  | 8 | 1 | 1.0625 | 1 | 2 | 1.125 |  |  |
| *A. baumannii* AB1617* | 32 | 0.25 | 0.375 | 4 | 0.25 | 0.375 | 16 | 2 |
|  | 16 | 0.5 | 0.375 | 2 | 1 | 0.75 |  |  |
|  | 8 | 1 | 0.5625 | 1 | 2 | 1.125 |  |  |

*: Clinical isolates. MaiA: maipomycin A; CLS: colistin. FICI: fractional inhibitory concentration index, FICI_A combined with B_ = FIC_A_+ FIC_B_ = [M(B)IC_A (combined)_/M(B)IC_A (alone)_] + [M(B)IC_B (combined)_/M(B)IC_B (alone)_]; Additivity (0.5< FICI≤1), Indifference (1< FICI≤2), Synergy (FICI≤0.5).

**Supplementary Table 11.** Two iron chelators increase colistin efficacy against *A. baumannii* ATCC19606

| Compounds | MIC (μg/mL) in combination | |  | MBIC (μg/mL) in combination | |  | MBIC (μg/mL) alone | |
| --- | --- | --- | --- | --- | --- | --- | --- | --- |
|  | Comp. | CLS | FICI | Comp. | CLS | FICI | Comp. | CLS |
| ColA | 16 | 0.125 | 0.3125 | 2 | 0.25 | 0.625 | 4 | 2 |
|  | 8 | 0.25 | 0.25 | 1 | 1 | 0.75 |  |  |
|  | 4 | 0.5 | 0.3125 | 0.5 | 1 | 0.625 |  |  |
| 2DP | 16 | 1 | 0.75 | 4 | 0.5 | 0.5625 | 64 | 1 |
|  | 8 | 1 | 0.625 | 2 | 0.5 | 0.53125 |  |  |
|  | 4 | 1 | 0.5625 | 1 | 1 | 1.015625 |  |  |

Comp: Compounds. ColA: collismycin A; 2DP: 2,2′-dipyridyl; CLS: colistin. FICI: fractional inhibitory concentration index, FICI_A combined with B_ = FIC_A_+ FIC_B_ = [M(B)IC_A (combined)_/M(B)IC_A (alone)_] + [M(B)IC_B (combined)_/M(B)IC_B (alone)_]; Additivity (0.5< FICI≤1), Indifference (1< FICI≤2), Synergy (FICI≤0.5).

**References**

1 Tsuge, N., Furihata, K., Shin-Ya, K., Hayakawa, Y., and Seto, H. (1999). Novel antibiotics pyrisulfoxin A and B produced by *Streptomyces californicus*. J Antibiot (Tokyo) 52(5), 505-507. doi: 10.7164/antibiotics.52.505.
